# Nitrogen-doped hierarchical porous carbons derived from biomass for oxygen reduction reaction

**DOI:** 10.3389/fchem.2023.1218451

**Published:** 2023-06-16

**Authors:** Min Wang, Yao Chen, Shunsheng Zhao, Cenkai Zhao, Guanxiong Wang, Mingbo Wu

**Affiliations:** ^1^ College of New Energy, China University of Petroleum (East China), Qingdao, China; ^2^ State Key Laboratory of Heavy Oil Processing, School of Chemical Engineering, China University of Petroleum (East China), Qingdao, China; ^3^ Shenzhen Academy of Aerospace Technology, Shenzhen, China

**Keywords:** biomass, hierarchical porous carbons, oxygen reduction, acidic medium, fuel cell

## Abstract

Nowadays biomass has become important sources for the synthesis of different carbon nanomaterials due to their low cost, easy accessibility, large quantity, and rapid regeneration properties. Although researchers have made great effort to convert different biomass into carbons for oxygen reduction reaction (ORR), few of these materials demonstrated good electrocatalytical performance in acidic medium. In this work, fresh daikon was selected as the precursor to synthesize three dimensional (3D) nitrogen doped carbons with hierarchical porous architecture by simple annealing treatment and NH_3_ activation. The daikon-derived material Daikon-NH_3_-900 exhibits excellent electrocatalytical performance towards oxygen reduction reaction in both alkaline and acidic medium. Besides, it also shows good durability, CO and methanol tolerance in different electrolytes. Daikon-NH_3_-900 was further applied as the cathode catalyst for proton exchange membrane (PEM) fuel cell and shows promising performance with a peak power density up to 245 W/g.

## Introduction

Nowadays, human beings are facing serious environmental problem like excessive CO_2_ emission and energy crisis. In order to solve these problems, researchers have made great effort to develop efficient and effective techniques for energy conversion and storage such as fuel cells, supercapacitors, lithium ion batteries and so on. In particular, fuel cells can utilize clean energy hydrogen (Hao et al., 2022; Zhang et al., 2022) and oxygen as gas fuels to convert chemical energy to electricity and heat without generating pollutants or CO_2_ emission. Typically, the hydrogen is oxidized at the anode and the oxygen is reduced at the cathode during the working of fuel cells. The kinetics of oxygen reduction reaction (ORR) is quite slow (Guo et al., 2022; Zaman and Chen, 2023) and Pt/C has been used as the commercial electrocatalyst for ORR (Zaman et al., 2021). However, Pt/C catalysts still suffers from some disadvantages including their high cost, and susceptible to the methanol/CO poisoning effect (Guo et al., 2022; Zhang et al., 2023). Therefore, it is necessary and important to develop novel cost-effective, high-performance ORR catalysts to replace the commercial Pt/C catalysts for practical applications in fuel cells.

Carbon nanomaterials (Yan et al., 2023) have attracted worldwide attention since they have demonstrated great potential in energy storage (Fu et al., 2023; Gadipelli et al., 2023; Gao et al., 2023; Mo et al., 2023; Senokos et al., 2023) and conversion (Zhang et al., 2022; Kim et al., 2023; Lv et al., 2023; Peng et al., 2023) to relieve the environmental problem and energy crisis (Gao et al., 2022; Marangon et al., 2023; Yan et al., 2023). Carbon nanomaterials also play a significant role in the field of electrocatalysis of ORR due to their low cost, high performance and excellent stability (Zaman et al., 2022a). For instance, N-doped carbon nanotubes (Ma et al., 2022; Choi et al., 2023) and N-doped graphene (Zaman et al., 2022b; Li et al., 2023) have been demonstrated to exhibit good ORR performance in alkaline medium. According to previous work (Shui et al., 2015; Guo et al., 2022; Ma et al., 2022; Yang et al., 2022; Li et al., 2023; Peng et al., 2023), it has been demonstrated that heteroatom doping of nitrogen is an effective way to improve the ORR electrocatalytic activity of carbon nanomaterials (Li et al., 2022). Apart from the heteroatom doping, the structure of the materials (Yu and Wuttig, 2023) also greatly affect the electronic and chemical properties, catalysis abilities and so on. Carbon materials with hierarchical porous structures are also extremely promising for energy storage and conversion. The porosity is a critical factor that affects the properties of the nanocarbons. According to the pore size, microporous carbons are defined as porous carbon materials (PCMs) with pore size smaller than 2 nm in diameter, mesoporous carbons are PCMs with pore size in the range of 2–50 nm, and macroporous carbons are PCMs with pore size larger than 50 nm (Wu et al., 2012). Hierarchical porous carbons (HPCs), a new family of PCMs, can be considered as carbon materials with multi-level pores. Comparing to the conventional PCMs, HPCs have a unique hierarchical porosity which allows to achieve the minimized diffusive resistance to mass transport from macropores and the high surface area for active site dispersion from micropores or mesopores. From the aspect of material structure, HPCs effectively construct a network of micropores, mesopores and/or macropores, utilizing the advantages of each scale pores with a synergistic effect to efficiently enhance their performances in catalysis (Jiao et al., 2023), energy storage (Wang T. et al., 2022a), adsorption and so on (Wang Y. et al., 2022b).

In recent years, biomass has become important sources for the synthesis of porous carbon nanomaterials due to their low cost, easy accessibility, large quantity, rapid regeneration and simple processing (Song et al., 2022; Han F. et al., 2023a; Han T. et al., 2023b; Chen et al., 2023; He et al., 2023; Jiao et al., 2023). Although researchers have made great effort to convert different biomass into porous carbon materials for ORR, few of these materials achieved good electrocatalytical performance in acidic medium (Wang et al., 2013; Chen et al., 2014; Guo et al., 2014; Pan et al., 2014; Guo et al., 2015; Liang et al., 2015). Thus, it still remains great challenges to develop novel high-performance oxygen reduction electrocatalysts from natural biomass. In this work, fresh daikon was selected as the precursor to synthesize three dimensional (3D) nitrogen-doped carbons with hierarchical porous architecture by simple annealing treatment and NH_3_ activation. The daikon-derived material Daikon-NH_3_-900 exhibits excellent electrocatalytical performance towards oxygen reduction reaction in both alkaline and acidic medium. Daikon-NH_3_-900 was further applied as the cathode catalyst for proton exchange membrane (PEM) fuel cell and shows promising performance with a peak power density up to 245 W/g.

## Experimental section

### Preparation of daikon-derived carbons

The raw material fresh daikon was purchased from the local supermarket and all the experiment data were collected from the same daikon.

The fresh daikon was peeled and cut into small slices. The daikon slices were then frozen dried by liquid nitrogen, and then placed into a lyophilizer at 10 mTorr for 3 days. The dried daikon slices were placed in a horizontal quartz tube of a furnace under argon gas (flow rate: 200 mL/min) at 900°C for 1 h, followed by NH_3_ activation (flow rate: 200 mL/min) for 15 min at the same temperature. For comparison, the daikon slices were annealed under argon gas (flow rate: 200 mL/min) at 900°C for 75 min without NH_3_ activation. The as-prepared samples were then washed by 1.0 M HCl for 12 h, noted as Daikon-NH_3_-900 and Daikon-Ar-900, respectively.

In this work, different annealing temperature of 800°C, 900°C, and 1,000°C were also investigated. The corresponding samples were noted as Daikon-NH_3_-800, Daikon-NH_3_-900 and Daikon-NH_3_-1000, respectively.

### Structure characterization

SEM images were taken using high resolution field emission scanning electron microscopy FEI Nova Nanolab200. TEM images were taken by transmission electron microscopy FEI Tecnai TF20 FEG. XRD was carried out on a Miniflex Desktop X-ray Diffractometer. XPS was conducted on VG Microtech ESCA 2000 using a monochromic Al X-ray source (97.9 W, 93.9 eV). The Raman spectra were collected by the Raman spectroscopy (Renishaw), using 514 nm laser. Thermogravimetric Analysis was performed on TGA (TA instrument Q50) with a heating rate of 10°C/min in air condition. Nitrogen adsorption isotherms were measured at −196°C on TriStar II 3020 Version 2.00 volumetric adsorption analyzers manufactured by Micromeritics. Before adsorption measurements, each sample was degassed under vacuum for 24 h at 150°C. The specific surface area of the samples was calculated using the Brunauer–Emmett–Teller (BET) method within the relative pressure range of 0.02–0.30. Incremental pore size distributions were obtained from the nitrogen adsorption isotherms by the Dollimore-Heal method provided by Micromeritics.

### Electrochemical characterization

Electrochemical measurements were performed on an electrochemical workstation (CHI760C, CH Instrument, United States) with a three-electrode electrochemical cell. All of the experiments were conducted at room temperature. A platinum wire was used as the counter electrode, and a silver/silver chloride electrode (Ag/AgCl) and saturated calomel electrode (SCE) were used as reference electrodes in O_2_ saturated 0.5 M H_2_SO_4_ and 0.1 M KOH electrolytes, respectively. The catalyst was drop casting on the glass carbon, followed by casting with a Nafion solution (0.05 wt% in ethanol) as the binder. The loading amount of the catalyst was about 250 μg/cm^2^.

The ORR activity of the electrocatalysts was evaluated by cyclic voltammetry (CV) and linear sweep voltammetry (LSV) techniques on rotating disk electrodes (RDEs) in oxygen saturated electrolytes. Methanol, CO tolerance test and durability test (30,000 s) on Daikon-NH_3_-900 and Pt/C were conducted by the chronoamperometric technique at −0.3 V vs. SCE or Ag/AgCl in O_2_ saturated 0.1 M KOH or 0.5 M H_2_SO_4_, respectively.

The electrochemical capacitance measurements were carried out in a standard three-electrode electrochemical cell at room temperature using 1.0 M aqueous H_2_SO_4_ solution as the electrolyte. The catalysts coated glassy carbon was employed as the working electrode, and the reference and counter electrodes were Ag/AgCl, and platinum, respectively.

### Fabrication of membrane electrode assembly (MEA)

First, 15 mg cathode catalyst powders (Daikon-NH_3_-900 or Daikon-Ar-900) were dispersed in the mixture of 0.5 mL distilled H_2_O, 1.0 mL isopropanol and 300 mg 5 wt% Nafion solution by ultra sonication and stirring. Then the cathode catalyst ink was coated on the 5.0 cm^2^ cathode carbon paper (ElectroChem Inc, Carbon Micro-porous Layer (CMPL)) with a brush. Commercial Hispec 4100 Pt/C catalyst was used in the anode. The anode catalyst ink was prepared by the same method, and then brush coated on the anode carbon paper. The catalyst (Daikon-NH_3_-900 or Daikon-Ar-900) loadings in the cathode were 3.0 mg/cm^2^ and Pt loadings in the anodes were 1.0 mg/cm^2^, respectively. MEAs were fabricated by hot pressing the cathode, DuPont Nafion 211 membrane and the anode together under the pressure of 60lb cm^−2^ at 130°C for 2 min.

### Test of PEM fuel cell

The fuel cell performance was tested at a single cell system. Single fuel cell was assembled with the as-prepared MEAs. H_2_ and O_2_ were used as the fuel and oxidant with 30 psi in the test process. Fuel cell polarization plots were recorded using fuel cell test stations (Arbin Instruments, United States). Pure hydrogen and O_2_, humidified at 80°C, were purged to the anode and cathode, respectively, at flow rates of 300 mL min^−1^ (H_2_) and 500 mL min^−1^ (O_2_).

## Results and discussion

**FIGURE 1:**
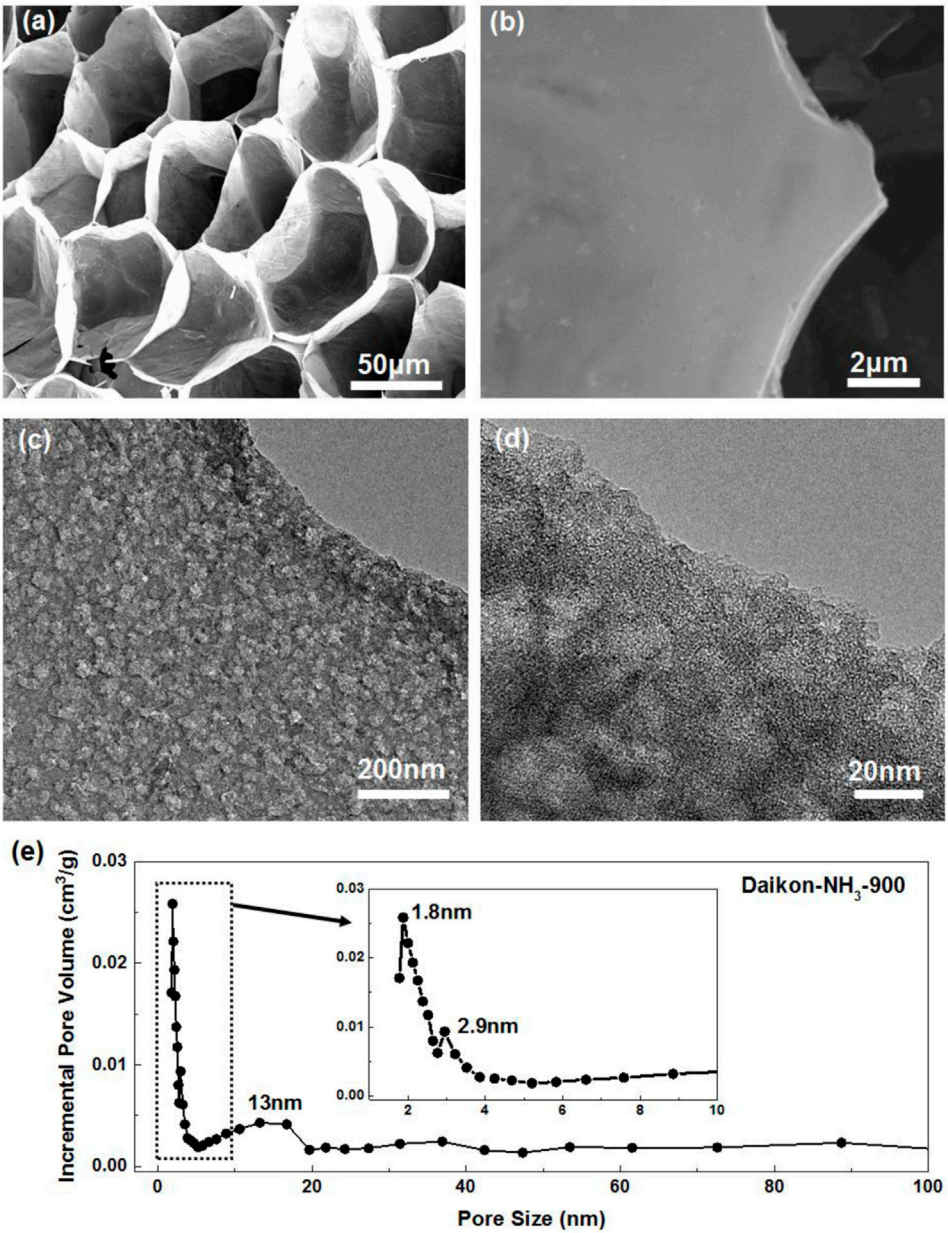
SEM images of **(A)** as-synthesized Daikon-NH_3_-900 without HCl washing treatment and **(B)** Daikon-NH_3_-900 after HCl washing treatment. **(C,D)** TEM images of Daikon-NH_3_-900 in different magnifications showing the holy nanostructure under different magnifications. **(E)** Pore size distribution of Daikon-NH_3_-900.

**FIGURE 2:**
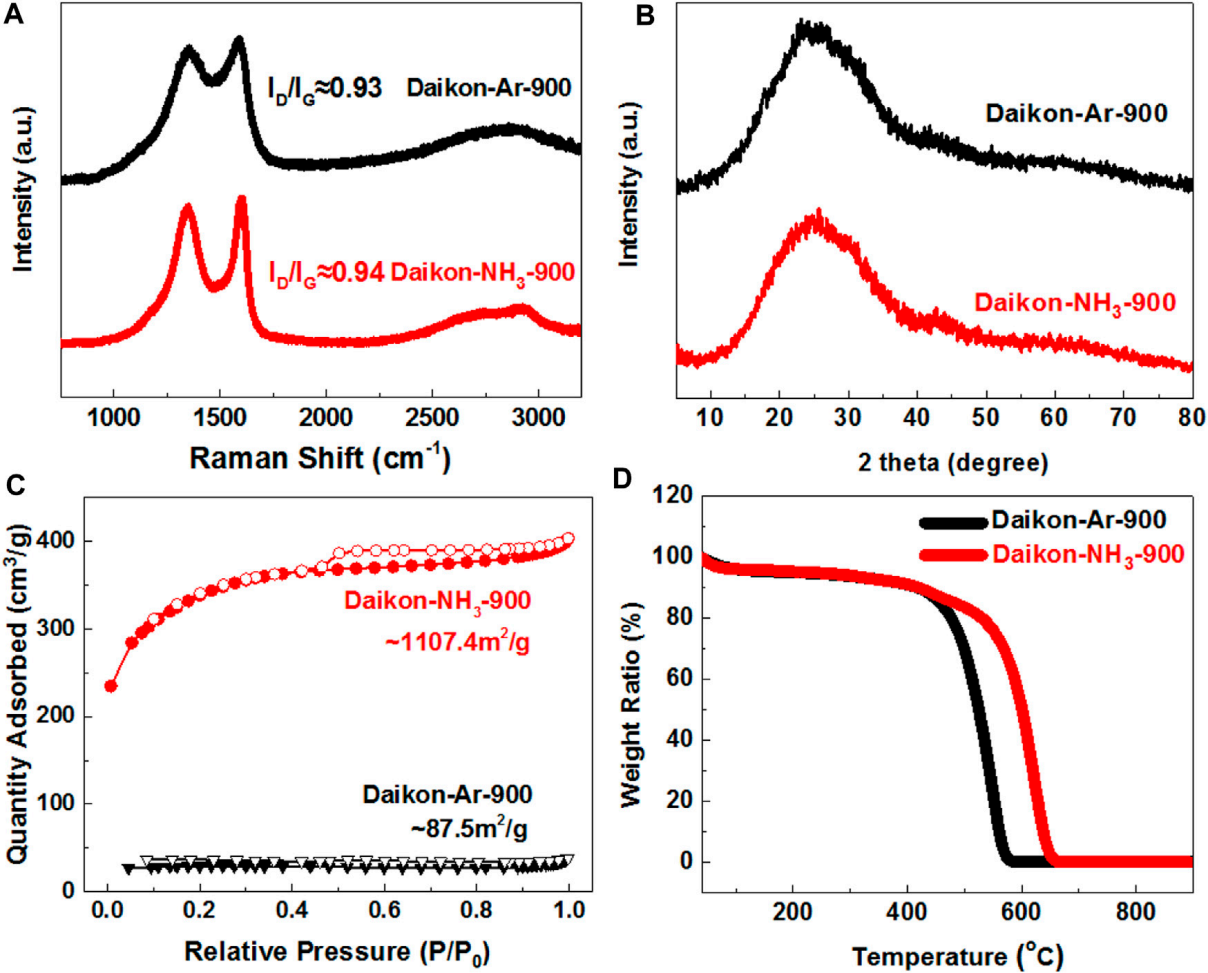
Structure characterizations of Daikon-NH_3_-900 and Daikon-Ar-900. **(A)** Raman spectra; **(B)** XRD patterns; **(C)** Nitrogen sorption isotherms and **(D)** Thermogravimetric analysis (TGA) in air condition.

The morphology of the porous carbons was first investigated by field emission scanning electron microscopy. The white dried daikon slices turns into black foam-like Daikon-NH_3_-900 after Ar annealing and NH_3_ activation as shown in Supplementary Figures S1A, B. The SEM images of as-synthesized sample without HCl treatment are presented in Figure 1A, Supplementary Figures S1C, D. As can be seen, Daikon-NH_3_-900 exhibits an interesting 3D porous network structure with uniform macro pores of ∼20 μm. These voids originate from the porous structure of the biomass materials daikon. The wall of these voids shown in Figure 1B is demonstrated to be hierarchical porous carbon sheets by further TEM investigation. Figures 1C, D; Supplementary Figures S2A, B presents the TEM images of Daikon-NH_3_-900 washed by HCl. As can be seen in the TEM images in Figure 1C, the carbon sheets of Daikon-NH_3_-900 process a highly porous structure. Abundant mesopores are uniformly distributed in the few carbon layers. Figure 1D with a higher magnification shows the average size of these mesopores ranges from 10 to 20 nm. These mesopores are mainly introduced by the ammonia activation. TEM images of Daikon-Ar-900 without ammonia activation are shown in Supplementary Figures S2C, D. The Daikon-Ar-900 is consisting of carbon sheets without porous structure, while Daikon-NH_3_-900 exhibits a hierarchical porosity as can be seen in Supplementary Figures S2A, B. The BET analysis further demonstrates the contribution of ammonia activation. According to Figure 2C, Daikon-Ar-900 has a very low specific surface area of ∼87.5 m^2^/g; surprisingly, the specific surface area of Daikon-NH_3_-900 is 1,107.4 m^2^/g which is about 12 times of Daikon-Ar-900. With the short time NH_3_ activation, the surface area of daikon-derived carbons is greatly increased by introducing large amounts of mesopores and micropores. Figure 1E shows the pore size distribution of Daikon-NH_3_-900. Apart from the macropores and mesopores, a large amount of micropores of ∼1.8 nm can be observed, resulting from the corrosive gas activation. Thus, the Daikon-NH_3_-900 is demonstrated to process a hierarchical porous structure consisting of macropores (∼20 μm), mesopores (3–20 nm) and micropores (∼1.8 nm) according to all the morphology investigations and BET analysis. The hierarchical porosity of Daikon-NH_3_-900 plays a great role in the energy storage and conversion applications. The abundant multi-level pores can effectively shorten the diffusion pathways and offer minimized diffusive resistance to mass transport on a large electrode/electrolyte interface. Besides, the hierarchical porosity can also offer rapid ion transport with improved rate capability. On the other hand, high porosity introduce defects and heteroatoms to further increase available active sites and effectively modulate their electronic and chemical characteristics.

The structure of Daikon-NH_3_-900 and Daikon-Ar-900 were then studied by Raman, XRD and TGA analysis as can be seen in Figures 2A, B, D, respectively. According to the XRD patterns in Figure 2B, both Daikon-NH_3_-900 and Daikon-Ar-900 show a broad C (002) peak at 26.3° and a weaker C (001) peak at 43.2°, indicating that the daikon have been successfully converted to graphitic carbons with amorphous domains during the carbonization process. The NH_3_ activation can further, etch the amorphous domains, resulting in a better graphitic crystal structure evidenced by Raman results. As shown in Figure 2A, Daikon-Ar-900 exhibits very broad D band at ∼1,356cm^−1^ and G band at 1,588cm^−1^. After NH_3_ activation, Daikon-NH_3_-900 shows well defined D band at ∼1,356cm^−1^ and G band at ∼1603cm^−1^, respectively. The observed redshift of G band in the Daikon-NH_3_-900 could be attributed to the nitrogen-doping effect via NH_3_ treatment (Das et al., 2008; Jorio, 2012). Compared to Daikon-Ar-900 (I_D_/I_G_ ≈ 0.93), the increasing ratio of D-band to G-band in the Daikon-NH_3_-900 (I_D_/I_G_ ≈ 0.94) is also related to the nitrogen-doping effect. Thus, simple NH_3_ activation step not only increases the surface area and porosity as discussed previously, but also introduces the nitrogen heteroatoms into the carbon planes of Daikon-NH_3_-900. Thermogravimetric Analysis (TGA) was employed to investigate the thermal stability of Daikon-NH_3_-900 and Daikon-Ar-900 in air condition. As can be seen in Figure 2D, both Daikon-NH_3_-900 and Daikon-Ar-900 show about 11% weight loss before 400°C, corresponding to the removal of oxygen-containing groups and moisture. Then a dramatic weight loss of the daikon-derived carbons at around 435°C can be observed. The Daikon-Ar-900 exhibits a decomposition temperature at around 581°C whereas the Daikon-NH_3_-900 decomposed at a higher temperature 653°C. The better thermal stability of Daikon-NH_3_-900 is probably associated with the higher graphitization and nitrogen doping effect. Both Daikon-NH_3_-900 and Daikon-Ar-900 exhibit 0.0 wt% after decomposition without any residues, demonstrating that they are metal-free materials.

**FIGURE 3:**
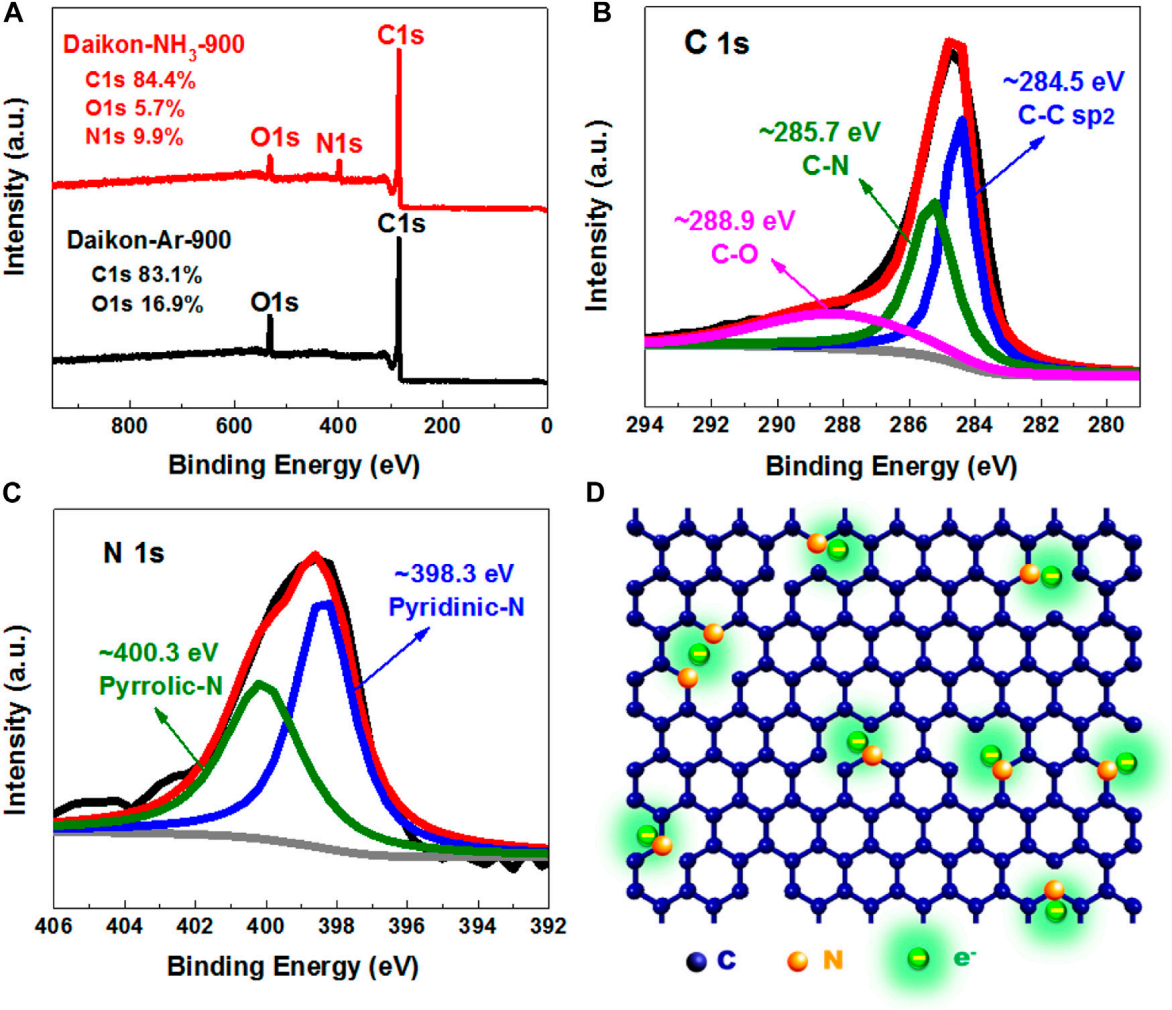
XPS analysis of Daikon-NH_3_-900. **(A)** XPS full spectrum of Daikon-NH_3_-900 and Daikon-Ar-900; **(B)** High resolution XPS C1s deconvoluted spectrum of Daikon-NH_3_-900; **(C)** High resolution XPS N1s deconvoluted spectrum of Daikon-NH_3_-900 and **(D)** Proposed chemical structure of Daikon-NH_3_-900.

To further study the elemental composition of Daikon-NH_3_-900, X-ray photoelectron spectroscopy (XPS) analysis was carried out. Figure 3A shows the survey spectra (0–950 eV) of Daikon-NH_3_-900 and Daikon-Ar-900. As can be seen, the XPS spectra include C 1s at ∼ 280 eV and O 1s at ∼ 533 eV with/without N 1s at ∼ 400 eV and no other impurities were detected. It is clear that NH_3_ activation successfully introduces nitrogen heteroatoms into Daikon-NH_3_-900 and the atomic percentage of N is as high as 9.9%. The high doping level of N in the Daikon-NH_3_-900 is closely related to the excellent electrochemical performance in the electrocatalysis and energy storage. To further investigate the state of C and N, high resolution XPS spectra of Daikon-NH_3_-900 were carried out. The high resolution XPS C 1s spectrum can be fitted into C-C (284.5 eV), C-N (285.7 eV) and C-O (288.9 eV) as shown in Figure 3B.

Consistently, the O 1s spectrum can be convoluted to two peaks of C-OH (533.4 eV) and O=C-OH 531.5 eV as can be seen in Supplementary Figure S3. For the high resolution N XPS spectrum in Figure 3C, the N 1s can be fitted into two main component peaks located at 398.3, and 400.3 eV, which could be assigned to pyridinic nitrogen and pyrrolic nitrogen, respectively. According to the XPS analysis, the proposed chemical structure of Daikon-NH3-900 is presented in Figure 3D. The electrons accumulate on the pyridinic and pyrrolic nitrogen atoms, because the N (*χ* = 3.04) has a higher electronegativity than that of C (*χ* = 2.55). The charge redistribution further changes the adsorption mode of O_2_ and facilitate the reduction of O_2_. The XPS results also demonstrates that both Daikon-NH_3_-900 and Daikon-Ar-900 are carbon materials without metal impurities, which is in good agreement with the previous TGA analysis.

With the understanding of the structure and composition, Daikon-NH_3_-900 with hierarchical porosity and rich nitrogen-doping can be considered as an excellent candidate for energy conversion and storage systems. Previously nitrogen-doped carbon nanomaterials have been demonstrated to process good ORR catalytic performance in alkaline medium such as 0.1 M KOH electrolyte. However, their ORR activities in acidic electrolyte were rarely reported which still remained challenges. Besides, to the best of our knowledge, none of the biomass-derived carbons show ORR activity in acid. In this work, for the first time, we investigated the ORR catalytic performance of daikon-derived 3D hierarchical porous carbons in the acidic medium.

**FIGURE 4:**
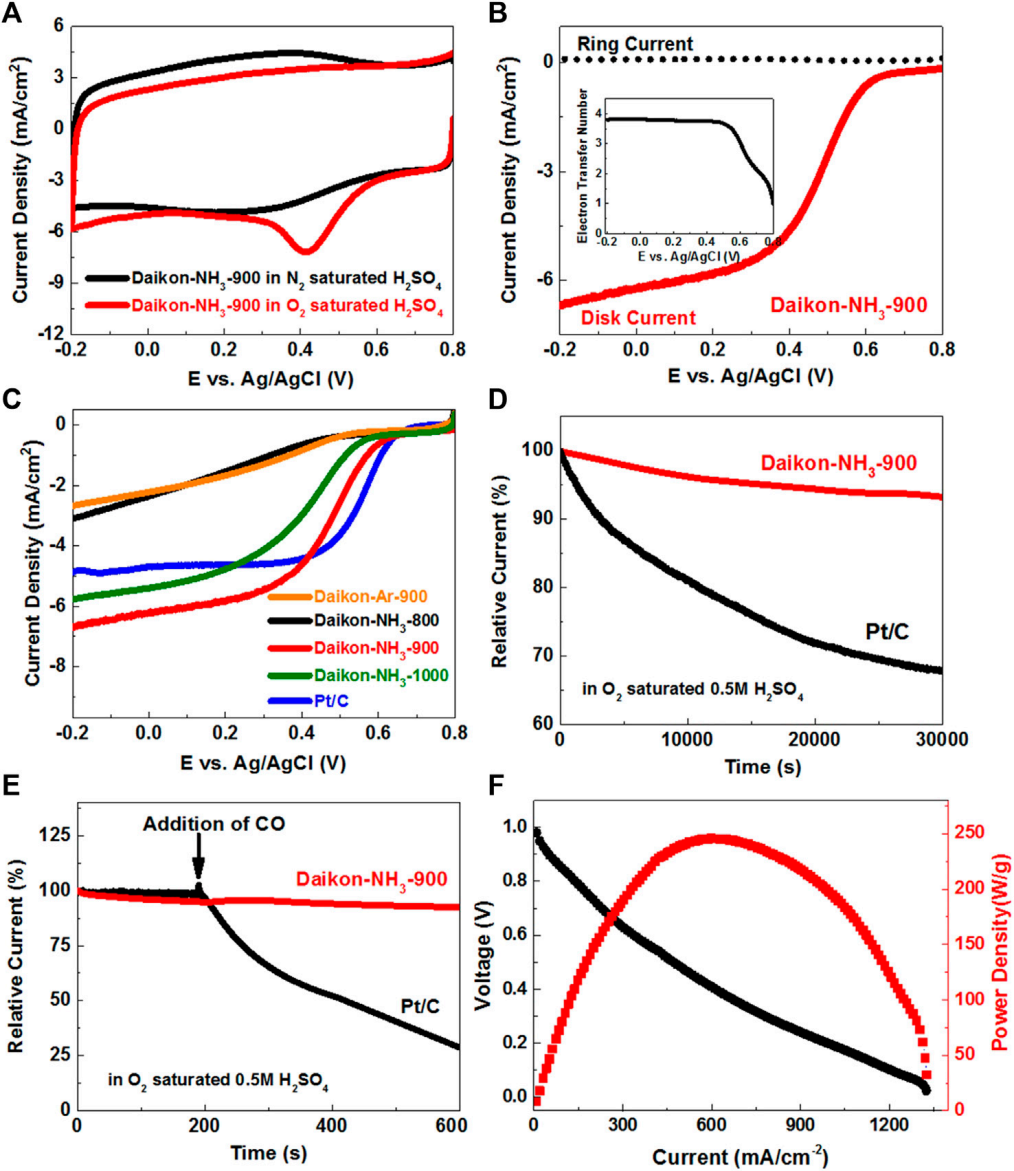
Catalytic activity towards electrochemical reduction of oxygen in acidic electrolyte at room temperature. **(A)** Cyclic voltammetry (CVs) of Daikon-NH_3_-900 in O_2_-saturated and N_2_-saturated 0.5 M H_2_SO_4_ obtained at a sweep rate of 50 mV s^−1^; **(B)** Linear sweep voltammograms (LSVs) of Daikon-NH_3_-900 on the RRDE at 1,600 rpm in 0.5 M O_2_-saturated H_2_SO_4_, the insert: electron transfer number of Daikon-NH_3_-900 estimated from the ring and disk currents; **(C)** LSVs of Daikon-Ar-900, Daikon-NH_3_-800, Daikon-NH_3_-900, Daikon-NH_3_-1000 and Pt/C electrodes in 0.5 M O_2_-saturated H_2_SO_4_ obtained at a sweep rate of 5 mV s^−1^ at 1,600 rpm; **(D)** Durability curves (i–t) of Daikon-NH_3_-900 and Pt/C obtained in at 0.3 V versus Ag/AgCl at a rotation rate of 1,000 rpm; **(E)** The current-time (i–t) chronoamperometric responses for ORR at the Daikon-NH_3_-900 and Pt/C electrodes in 0.5 M O_2_-saturated H_2_SO_4_ aqueous solution at 0.3 V versus Ag/AgCl, CO was added at around 200 s; **(F)** Polarization curve and power density of the MEA fabricated with of Daikon-NH_3_-900 (3.0 mg/cm^2^) as cathode electrode for H_2_/O_2_ at 80°C, DuPont Nafion 211 membrane, 30/30 psi anode and cathode back pressure. Anode electrode was Pt coated electrode with loading amount of 1.0 mg/cm^2^.

The ORR electrocatalytic activity of Daikon-NH_3_-900 was first tested by conventional three-electrode cyclic voltammetry (CV) in O_2_ and N_2_ saturated 0.5 M H_2_SO_4_ aqueous solutions, respectively. Figure 4A shows a characteristic ORR peak at about 0.40 V (vs. Ag/AgCl) for oxygen reduction on the Daikon-NH_3_-900 electrode in the O_2_ saturated electrolyte, but not in the N_2_ saturated electrolyte. To investigate the kinetics of ORR, we used rotation ring-disk electrode (RRDE) to evaluate ORR performance of the Daikon-NH_3_-900. As shown in Figure 4B, the ORR onset potential (E_onset_) and half-wave potential (E_1/2_) of the Daikon-NH_3_-900 is about 0.69V and 0.49 V (vs. Ag/AgCl), respectively. According to the current densities collected on the ring and disk, the electron transfer number was calculated to be about 4.0 as shown in the insert of Figure 4B (Luo et al., 2023; Wang et al., 2023). A series of linear sweep voltammograms (LSVs) for Daikon-NH_3_-900 were measured on a rotating disk electrode (RDE) from 400 to 1600 rpm, showing an increased current density with increasing rotations speeds in Supplementary Figure S4A, indicating an excellent electrocatalytical performance with desired four-electron ORR process in consistent with RRDE analysis. To better understand the excellent electrocatalytical activity of Daikon-NH_3_-900, the LSVs of Daikon-Ar-900 without NH_3_ activation was measured for comparison. As shown in Figure 4C, the Daikon-Ar-900 exhibits negligible ORR performance in 0.5 M H_2_SO_4_ with a poor E_1/2_ and extremely low current density. Thus, the electrocatalytical active sites of Daikon-NH_3_-900 should be considered as the nitrogen heteroatoms. The excellent ORR performance of Daikon-NH_3_-900 in the acidic medium is highly related to the high N doping level of 9.9 at%. After confirming the important influence of nitrogen doping from NH_3_ activation, different thermal annealing and activation temperatures including 800°C, 900°C, and 1000°C were selected to further optimize the ORR performance. Figure 4C; Supplementary Figures S4B, C present the electrochemical reduction behaviors of daikon derived carbons under different temperatures. According to Figure 4C, the LSVs in show an increased ORR onset potential and limited current density in the order of Daikon-NH_3_-800< Daikon-NH_3_-1000< Daikon-NH_3_-900, indicating that 900°C is the best reaction temperature for the carbonization of daikon. The corresponding ring current density and electron transfer number curves of daikon derived at different temperatures are shown in Supplementary Figures S4B, C. The different ORR catalytic performance of these daikon-derived carbons (Daikon-NH_3_-800, Daikon-NH_3_-900 and Daikon-NH_3_-1000) are caused by the structures as shown in Supplementary Figure S4D. According to the Raman spectra of Daikon-NH_3_-800, annealing treatment at 800°C of the daikon only results in a low graphitization, and this is responsible for the worst electrocatalytic performance. When increasing the thermal annealing temperature to 900°C and 1,000°C, the well-defied D-band and G-band indicate the good graphitic crystal structures of Daikon-NH_3_-900 and Daikon-NH_3_-1000. However, the low I_D_/I_G_ ratio (≈0.89) of Daikon-NH_3_-1000 indicates that the Daikon-NH_3_-1000 has a relatively low doping level probably due to the overheating. Thus, in the carbonization of the daikon, a modest annealing temperature of 900°C is appropriate for both graphitization and nitrogen doping, making Daikon-NH_3_-900 the best ORR electrocatalyst. It should also be noted that the ORR performance of the Daikon-NH_3_-900 is even comparable to commercial Pt/C in acidic electrolyte according to the LSVs in Figure 4C.

Figure 4D shows the stability of the Daikon-NH_3_-900 and Pt/C electrodes under a constant voltage of 0.3 V (vs. Ag/AgCl). It is obvious that the Daikon-NH_3_-900 exhibits excellent ORR stability with a very slow attenuation and a high current retention of 93.4% after 30,000 s, which is much better than that of the commercial Pt/C catalyst. The Daikon-NH_3_-900 was further tested by the possible methanol crossover and carbon monoxide poisoning effect. As shown in Figure 4E; Supplementary Figure S6A, the original cathodic ORR current of Daikon-NH_3_-900 under 0.3 V (vs. Ag/AgCl) does not show any significant changes after the addition of 3.0 M methanol or CO, suggesting that Daikon-NH_3_-900 is free from the methanol crossover effect and CO poisoning. In comparison, the commercial Pt/C suffers severe methanol crossover and CO poisoning problem in 0.5 M H_2_SO_4_.

Then, we further investigated the catalytic performance of the Daikon-NH_3_-900 in the proton exchange membrane fuel cell (PEMFC). The Daikon-NH_3_-900 and the Daikon-Ar-900 were applied as cathode catalyst and fabricated into the single cell, respectively. Figure 4F shows the device performance of the membrane electrode assemblies fabricated with the Daikon-NH_3_-900 (MEA-Daikon-NH_3_-900). MEA-Daikon-NH_3_-900 has a high open circuit voltage of ∼0.98 V. The peak power density is as high as ∼245 W/g and the limiting current density reaches ∼1303 mA/cm^2^. Supplementary Figures S7A–C present the performance comparison of MEA-Daikon-NH_3_-900 and MEA-Daikon-Ar-900. MEA-Daikon-NH_3_-900 shows a higher OCV and larger power density than that of MEA-Daikon-Ar-900 due to the nitrogen doping effect. The excellent device performance such as large limiting current and power density are attributed to the hierarchical porosity and rich heteroatom doping of Daikon-NH_3_-900.

**FIGURE 5:**
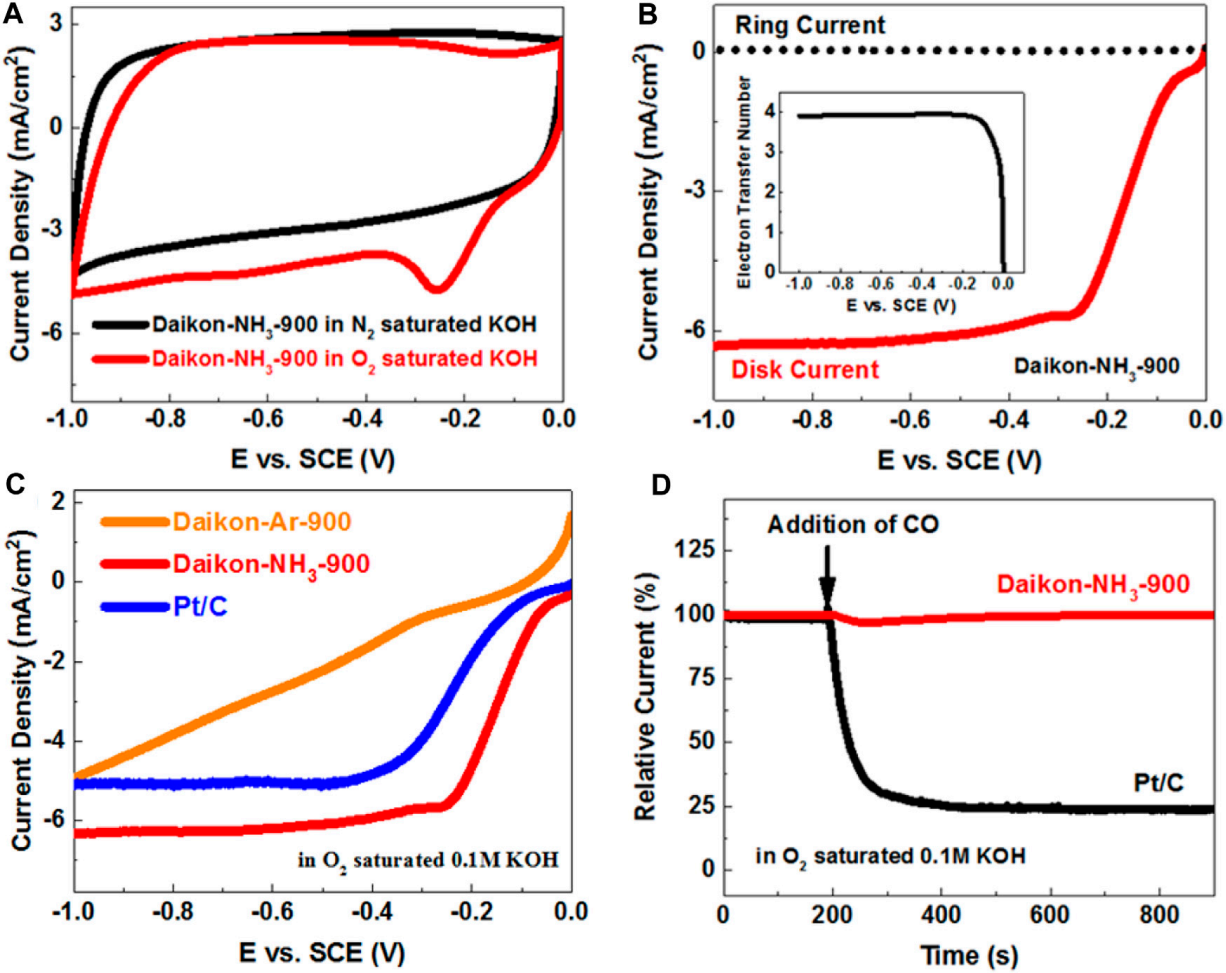
**(A)** CVs of Daikon-NH_3_-900 obtained at a sweep rate of 50 mV s^−1^ in O_2_- and N_2_-saturated 0.1 M KOH aqueous solution; **(B)** LSVs of Daikon-NH_3_-900 on the RRDE at 1600rpm in O_2_ saturated 0.1 M KOH, the insert: electron transfer number of Daikon-NH_3_-900 estimated from the ring and disk currents; **(C)** LSVs of Daikon-Ar-900, Daikon-NH_3_-900 and Pt/C obtained at a rotation rate of 1600rpm obtained at a sweep rate of 5 mV s^−1^ in 0.1 M O_2_-saturated KOH; **(D)** The current-time (i–t) chronoamperometric responses for ORR at the Daikon-NH_3_-900 and Pt/C electrodes in 0.1 M O_2_-saturated KOH aqueous solution at −0.3 V versus SCE, and CO was added at around 200 s.

The ORR electrocatalytical performance of the Daikon-NH_3_-900 in alkaline medium was then investigated using the typical electrolyte 0.1 M O_2_-saturated KOH. As shown in Figure 5A, a very strong ORR peak is observed at about −0.20 V (vs. SCE) on the Daikon-NH_3_-900 electrode in the O_2_ saturated KOH. The LSV and ORR kinetics of Daikon-NH_3_-900 was investigated on RRDE as shown in Figure 5B. The E_onset_ and E_1/2_ of Daikon-NH_3_-900 is 0.03 V vs. SCE and −0.14 V vs. SCE, respectively, while the electron transfer number is 4 as shown in the insert of Figure 5B. Figure 5C displays the LSVs of Daikon-NH_3_-900, Daikon-NH_3_-900 and commercial Pt/C electrodes at 1,600 rpm. As expected, the ORR performance of Daikon-Ar-900 is really inferior, and this is in good agreement with the results obtained in acidic electrolyte. The Daikon-NH_3_-900 with nitrogen doping exhibits even better ORR activity than Pt/C in alkaline electrolyte, showing more positive E_onset_ and E_1/2_. More electrochemical results of ORR are presented in Supplementary Figure S5. As can be seen in Supplementary Figure S5A, Daikon-NH_3_-900 achieves the diffusion limiting current plateau at a positive potential of 0.2 V vs. SCE, indicating a high density of electroactive sites for the reduction of oxygen. Supplementary Figures S5B–D presents LSV curves, the corresponding ring currents and electron transfer numbers of Daikon-NH_3_-800, Daikon-NH_3_-900, Daikon-NH_3_-1000, respectively. As expected, the Daikon-NH_3_-900 exhibits the best ORR performance, which is in consistent with the results in acidic medium. Daikon-NH_3_-800 with a poor graphitic structure shows the worst electrocatalytic activity towards ORR in basic electrolyte. It should be noted that the ORR performance of Daikon-NH_3_-1000 including the E_onset_, E_1/2_ and reduction current density is similar to that of Daikon-NH_3_-900, as can be seen in Supplementary Figure S5B. As discussed, the Daikon-NH_3_-1000 processes a similar graphitization to Daikon-NH_3_-900 with a lower nitrogen doping level. Compared to the ORR in acid, the catalytic performance of Daikon-NH_3_-1000 in alkaline is better. This is because that the nitrogen heteroatoms in the Daikon-NH_3_-1000 are sufficient to catalyze ORR in the KOH, while more electroactive sites are need for the acidic ORR process. However, as Supplementary Figure S5D showed, the electron transfer number of Daikon-NH_3_-900 is still higher than that of Daikon-NH_3_-1000 due to a higher density of nitrogen active sites, indicating a more efficient oxygen reduction process on the Daikon-NH_3_-900 electrode. As Figure 5D; Supplementary Figure S6B show, the Daikon-NH_3_-900 exhibits very good CO and methanol tolerance in alkaline medium, showing great advantages than the commercial Pt/C catalyst. The durability test was also conducted in 0.1 M O_2_ saturated KOH. According to Supplementary Figure S6C, the Daikon-NH_3_-900 shows excellent stability with the retention of 97.6% after 30,000 s, while Pt/C only had a retention of 70.7%. Daikon-NH_3_-900 exhibits outstanding long-term stability in both acidic and basic electrolytes. Daikon-NH_3_-900 has been demonstrated to be high-performance ORR electrocatalyst in acidic and alkaline medium on the three-electrode system.

## Conclusion

3D nitrogen-doped carbon materials with hierarchical porous architecture were derived from biomass by simple annealing treatment and NH_3_ activation. The daikon-derived material Daikon-NH_3_-900 exhibits excellent electrocatalytical performance towards oxygen reduction reaction in both alkaline and acidic medium. It shows very positive oxygen reduction onset and half-wave potential, excellent CO and methanol tolerance in different electrolytes. Daikon-NH_3_-900 was further applied as the cathode catalyst for proton exchange membrane (PEM) fuel cell and shows promising performance with a peak power density up to 245 W/g, demonstrating excellent performance among ORR electrocatalyst made from biomass.

## Data Availability

The original contributions presented in the study are included in the article/Supplementary Material, further inquiries can be directed to the corresponding authors.

## Supplementary material

SUPPLEMENTARY FIGURE S1
**(A)** Photography of freeze-dried daikon slices; **(B)** Photography of as-synthesized Daikon-NH_3_-900; **(C,D)** SEM images of as-synthesized Daikon-NH_3_-900.

SUPPLEMENTARY FIGURE S2 TEM images of **(A,B)** Daikon-NH_3_-900 and **(C,D)** Daikon-Ar-900 after HCl washing.

SUPPLEMENTARY FIGURE S3 High resolution XPS O1s deconvoluted spectrum of Daikon-NH_3_-900.

SUPPLEMENTARY FIGURE S4
**(A)** LSVs of Daikon-NH_3_-900 at different rotating speeds (400, 600, 900, 1,200, and 1,600 rpm) at a sweep rate of 5 mV s^−1^ in 0.5 M O_2_-saturated H_2_SO_4_; **(B)** The electron transfer number and **(C)** The ring current of Daikon-NH_3_-800, Daikon-NH_3_-900 and Daikon-NH_3_-1000 electrodes in 0.5 M O_2_-saturated H_2_SO_4_ obtained on RRDE at a rotation speed of 1,600 rpm; **(D)** Raman spectra of Daikon-NH_3_-800, Daikon-NH_3_-900 and Daikon-NH_3_-1000.

SUPPLEMENTARY FIGURE S5
**(A)** LSVs of Daikon-NH_3_-900 at different rotating speed (400, 900, 1,200, and 1,600 rpm) at a sweep rate of 5 mV s^−1^ in 0.1 M O_2_-saturated KOH aqueous solution. **(B)** RRDE LSVs of Daikon-NH_3_-800, Daikon-NH_3_-900 and Daikon-NH_3_-1000 obtained at a rotation rate of 1600rpm in 0.1 M O_2_-saturated KOH; **(C)** The corresponding ring current of Supplementary Figure S4B; **(D)** The electron transfer number of Daikon-NH_3_-800, Daikon-NH_3_-900 and Daikon-NH_3_-1000 estimated from Supplementary Figures S4B, C.

SUPPLEMENTARY FIGURE S6
**(A)** The current-time (i-t) chronoamperometric responses for ORR at the Daikon-NH_3_-900 and Pt/C electrodes in 0.5 M O_2_-saturated H_2_SO_4_ aqueous solution at 0.3 V vs. Ag/AgCl, 3.0 M methanol was added at around 200 s; **(B)** The current-time (i-t) chronoamperometric responses for ORR at the Daikon-NH_3_-900 and Pt/C electrodes in 0.1 M O_2_-saturated KOH aqueous solution at −0.3 V vs. SCE, and 3.0 M methanol was added at around 200 s; **(C)** Durability curves (i-t) of Daikon-NH_3_-900 and Pt/C obtained in at −0.3 V vs. SCE at a rotation rate of 1000 rpm.

SUPPLEMENTARY FIGURE S7
**(A)** Polarization curve and **(B)**. Power density of the MEAs fabricated with of Daikon-NH_3_-900 (3.0 mg/cm^2^) and Daikon-Ar-900 (3.0 mg/cm^2^) as cathode electrodes for H_2_/O_2_ at 80°C, DuPont Nafion 211 membrane, 30/30 psi anode and cathode back pressure. Anode electrodes were Pt coated electrode with loading amount of 1.0 mg/cm^2^; **(C)**. Summary of the experimental data in Figure 5.11 **(A,B)**.
