# Supplementary material for: Nitrogen-doped hierarchical porous carbons derived from biomass for oxygen reduction reaction
Source: Front Chem. 2023 Jun 16;11:1218451. doi: 10.3389/fchem.2023.1218451 (PMC10311552; doi:10.3389/fchem.2023.1218451)
Supplement: Supplementary file 1 [file Presentation1.pdf]

Supplementary material

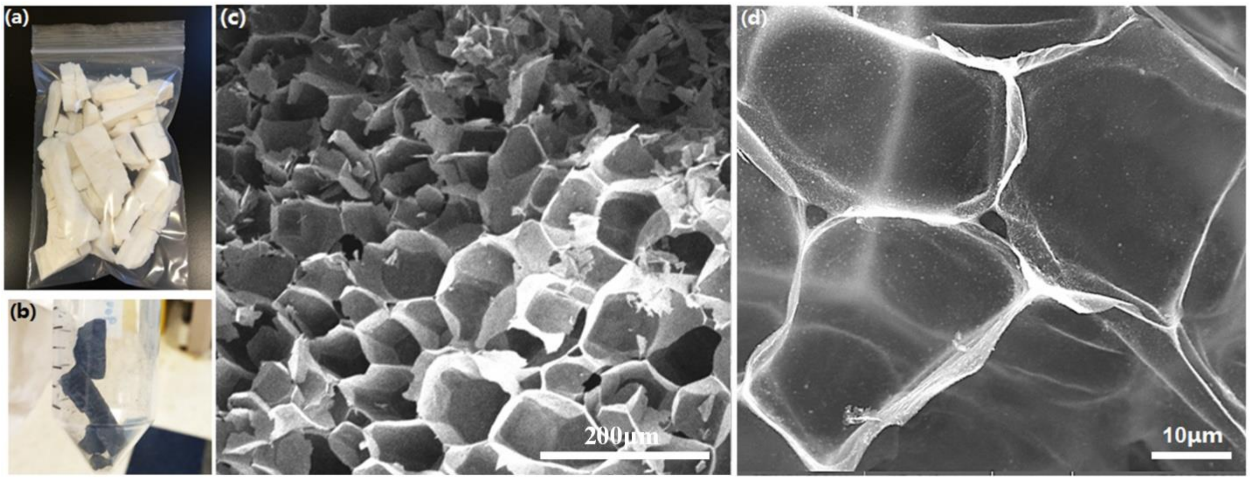

Figure S1 (a) Photography of freeze dried daikon slices; (b). Photography of as-synthesized Daikon-NH_3-900; (c), (d). SEM images of as-synthesized Daikon-NH_3-900.

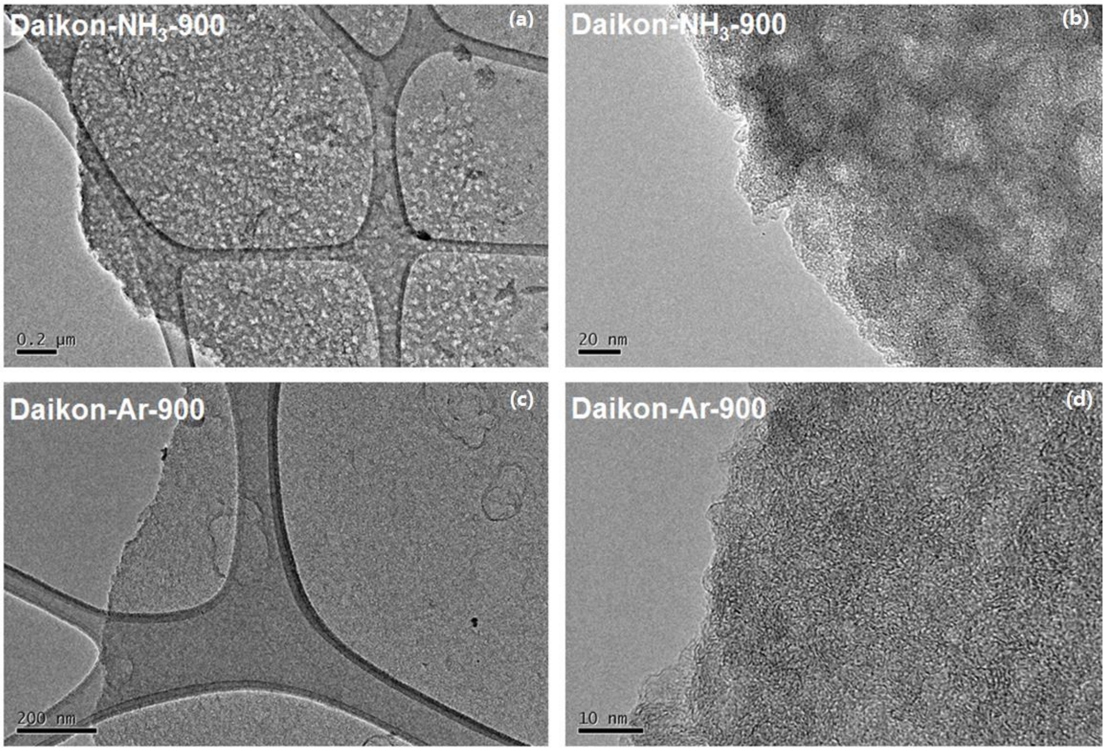

Figure S2. TEM images of (a), (b) Daikon-NH_3-900 and (c), (d) Daikon-Ar-900 after HCl washing.

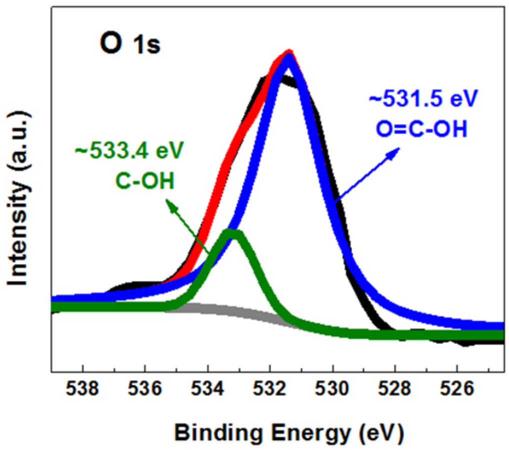

Figure S3. High resolution XPS O1s deconvoluted spectrum of Daikon-NH_3-900.

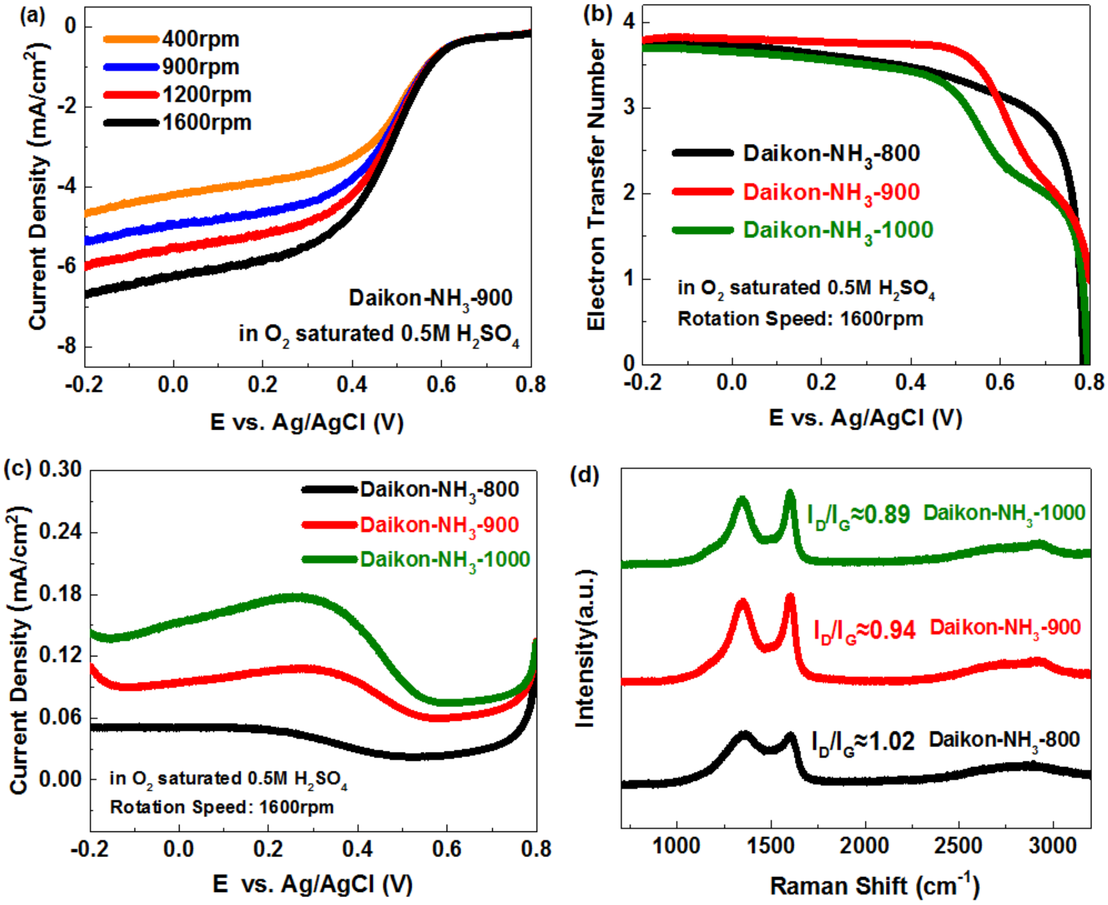

Figure S4 (a). LSVs of Daikon-NH_3-900 at different rotating speeds (400rpm, 600rpm, 900rpm, 1200rpm and 1600rpm) at a sweep rate of 5 mV s^{-1} in 0.5M O_2-saturated H_2SO_4; (b) The electron

transfer number and (c). The ring current of Daikon-NH_3-800, Daikon-NH_3-900 and Daikon-NH_3-1000 electrodes in 0.5M O_2-saturated H_2SO_4 obtained on RRDE at a rotation speed of 1600 rpm; (d). Raman spectra of Daikon-NH_3-800, Daikon-NH_3-900 and Daikon-NH_3-1000.

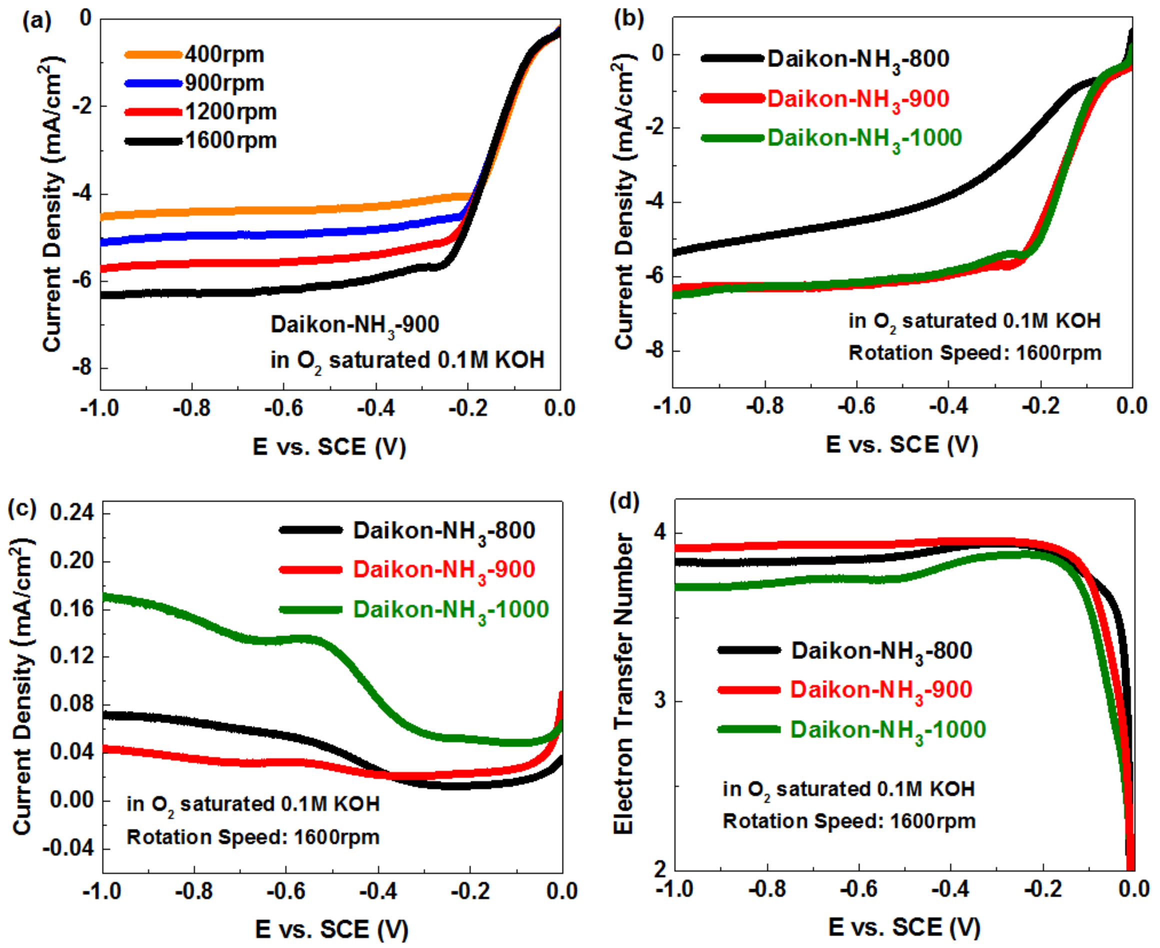

Figure S5 (a). LSVs of Daikon-NH_3-900 at different rotating speed (400rpm, 900rpm, 1200rpm and 1600rpm) at a sweep rate of 5 mV s^{-1} in 0.1M O_2-saturated KOH aqueous solution. (b). RRDE LSVs of Daikon-NH_3-800, Daikon-NH_3-900 and Daikon-NH_3-1000 obtained at a rotation rate of 1600rpm in 0.1M O_2-saturated KOH; (c). The corresponding ring current of Figure S4 (b); (d). The electron transfer number of Daikon-NH_3-800, Daikon-NH_3-900 and Daikon-NH_3-1000 estimated from Figure S4 (b) and (c).

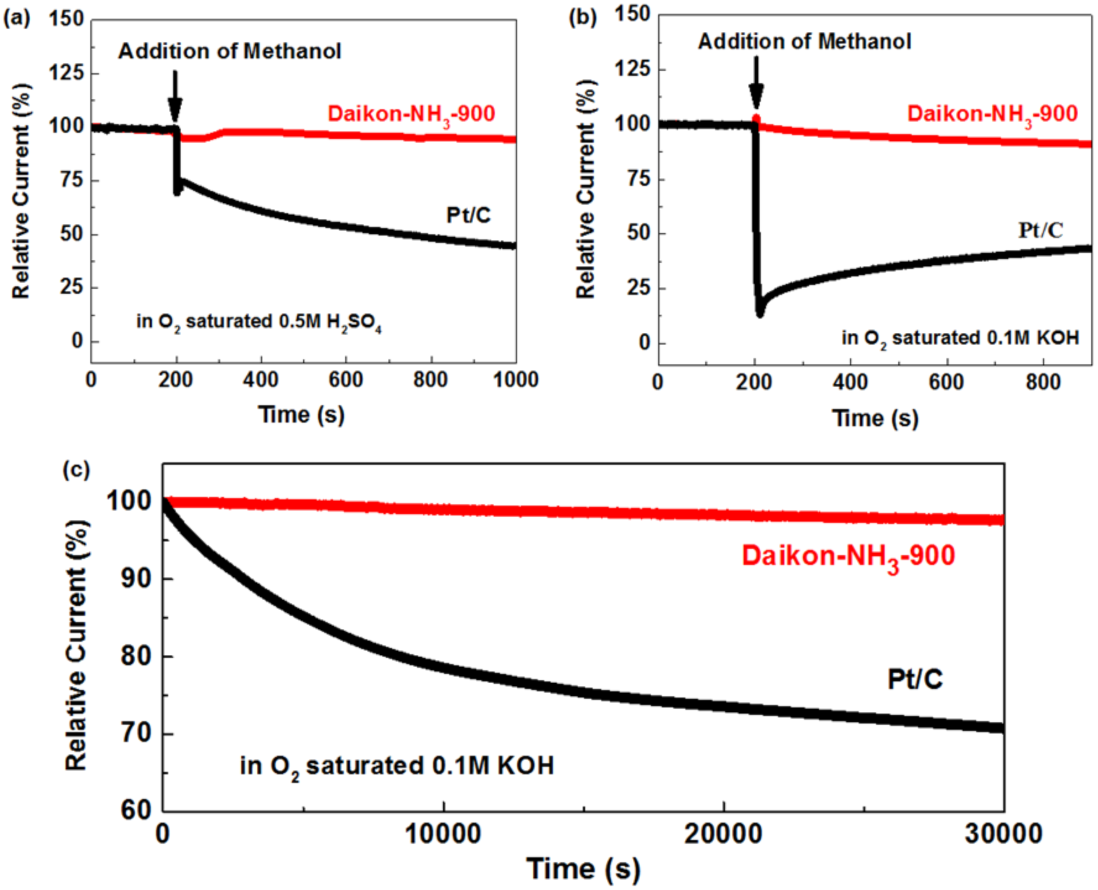

Figure S6 (a). The current-time (i-t) chronoamperometric responses for ORR at the Daikon-NH_3-900 and Pt/C electrodes in 0.5M O_2-saturated H_2SO_4 aqueous solution at 0.3 V versus Ag/AgCl, 3.0 M methanol was added at around 200 s; (b). The current-time (i-t) chronoamperometric responses for ORR at the Daikon-NH_3-900 and Pt/C electrodes in 0.1M O_2-saturated KOH aqueous solution at -0.3 V versus SCE, and 3.0 M methanol was added at around 200 s; (c). Durability curves (i-t) of Daikon-NH_3-900 and Pt/C obtained in at -0.3V versus SCE at a rotation rate of 1000 rpm.

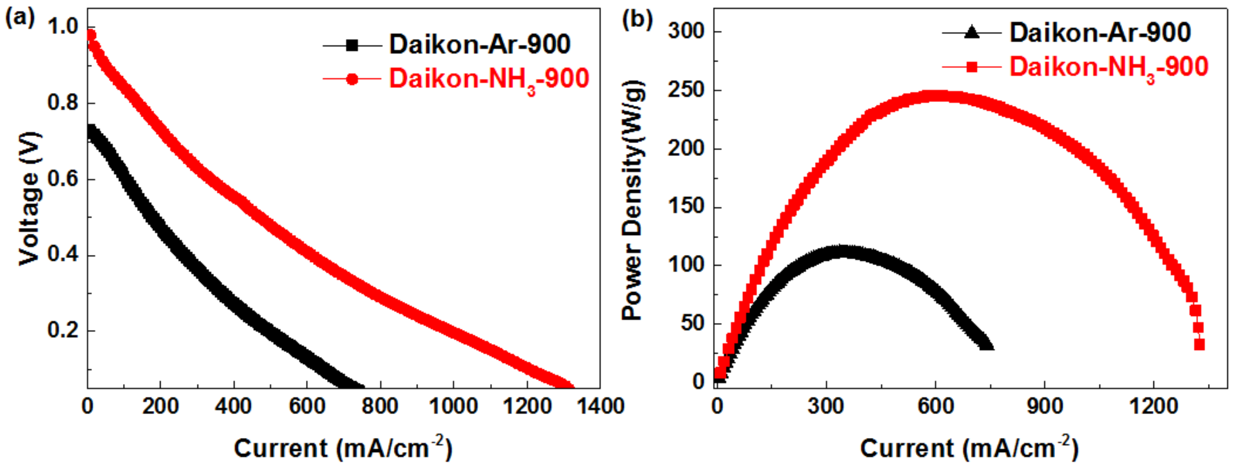

(c)

	Daikon-NH_3-900	Daikon-Ar-900
**Open Circuit Voltage**	0.98V	0.73V
**Limiting Current Density**	1303 mA/cm^2	730 mA/cm^2
**Peak Power Density**	245 W/g	110 W/g

Figure S7 (a). Polarization curve and (b). Power density of the MEAs fabricated with of Daikon-NH_3-900 (3.0 mg/cm^2) and Daikon-Ar-900 (3.0 mg/cm^2) as cathode electrodes for H_2/O_2 at 80˚C, DuPont Nafion 211 membrane, 30/30 psi anode and cathode back pressure. Anode electrodes were Pt coated electrode with loading amount of 1.0 mg/cm^2; (c). Summary of the experimental data in Figure 5.11 (a) and (b).
